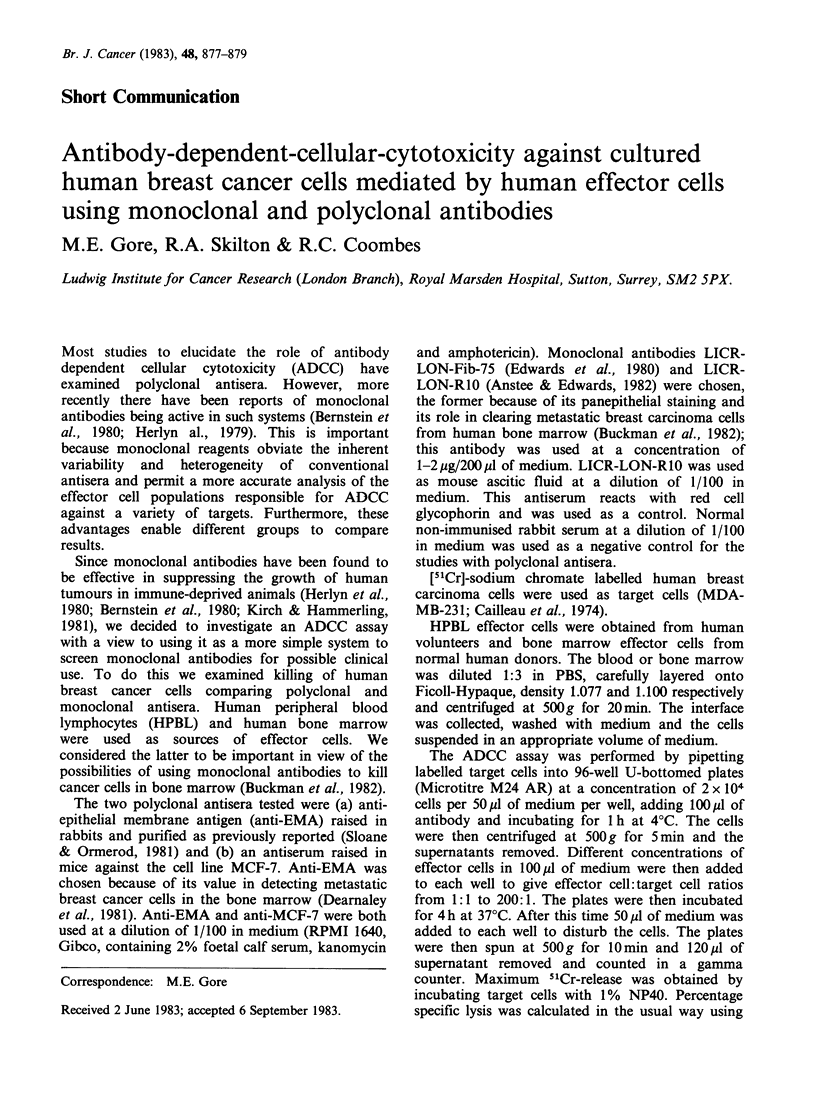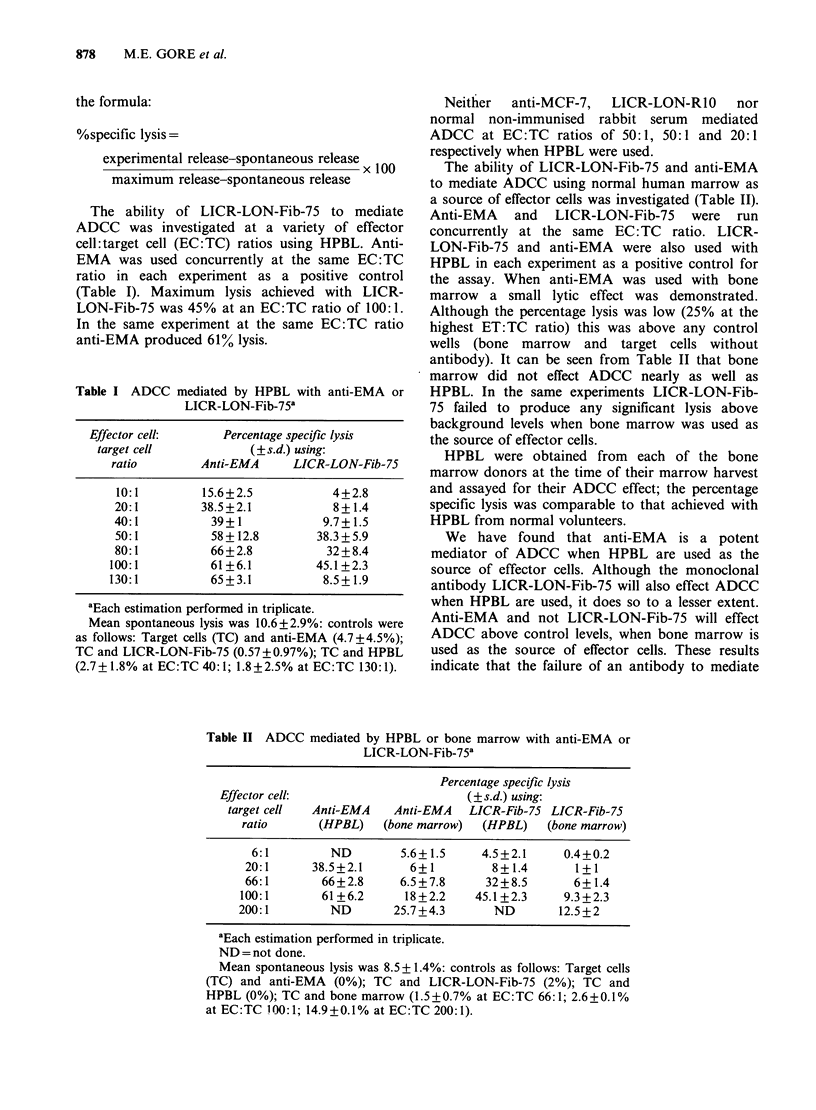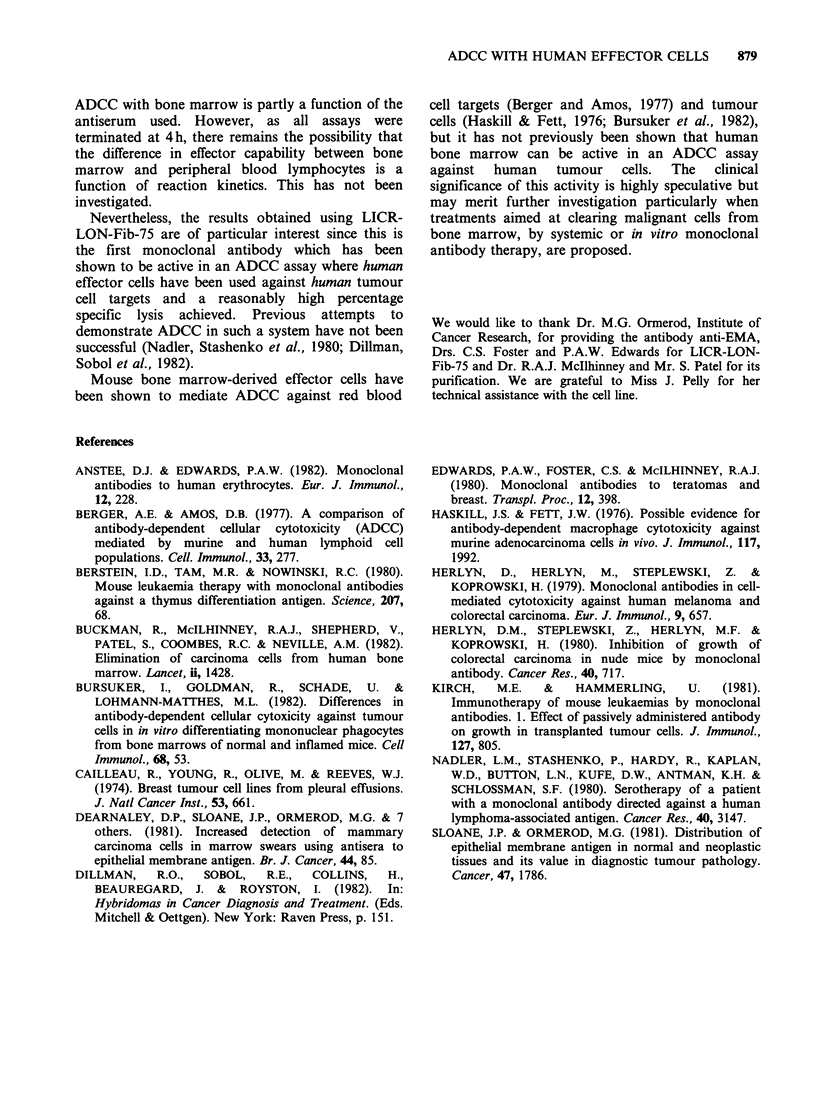# Antibody-dependent-cellular-cytotoxicity against cultured human breast cancer cells mediated by human effector cells using monoclonal and polyclonal antibodies.

**DOI:** 10.1038/bjc.1983.282

**Published:** 1983-12

**Authors:** M. E. Gore, R. A. Skilton, R. C. Coombes


*Br. J. Cancer* (1983), **48**, 877–879

### Short Communication

### Antibody-dependent-cellular-cytotoxicity against cultured human breast cancer cells mediated by human effector cells using monoclonal and polyclonal antibodies

M.E. Gore, R.A. Skilton & R.C. Coombes

*Ludwig Institute for Cancer Research (London Branch), Royal Marsden Hospital, Sutton, Surrey, SM2 5PX.*

Most studies to elucidate the role of antibody dependent cellular cytotoxicity (ADCC) have examined polyclonal antisera. However, more recently there have been reports of monoclonal antibodies being active in such systems (Bernstein *et al.*, 1980; Herlyn al., 1979). This is important because monoclonal reagents obviate the inherent variability and heterogeneity of conventional antisera and permit a more accurate analysis of the effector cell populations responsible for ADCC against a variety of targets. Furthermore, these advantages enable different groups to compare results.

Since monoclonal antibodies have been found to be effective in suppressing the growth of human tumours in immune-deprived animals (Herlyn *et al.*, 1980; Bernstein *et al.*, 1980; Kirch & Hammerling, 1981), we decided to investigate an ADCC assay with a view to using it as a more simple system to screen monoclonal antibodies for possible clinical use. To do this we examined killing of human breast cancer cells comparing polyclonal and monoclonal antisera. Human peripheral blood lymphocytes (HPBL) and human bone marrow were used as sources of effector cells. We considered the latter to be important in view of the possibilities of using monoclonal antibodies to kill cancer cells in bone marrow (Buckman *et al.*, 1982).

The two polyclonal antisera tested were (a) anti-epithelial membrane antigen (anti-EMA) raised in rabbits and purified as previously reported (Sloane & Ormerod, 1981) and (b) an antiserum raised in mice against the cell line MCF-7. Anti-EMA was chosen because of its value in detecting metastatic breast cancer cells in the bone marrow (Dearnaley *et al.*, 1981). Anti-EMA and anti-MCF-7 were both used at a dilution of 1/100 in medium (RPMI 1640, Gibco, containing 2% foetal calf serum, kanomycin and amphotericin). Monoclonal antibodies LICR-LON-Fib-75 (Edwards *et al.*, 1980) and LICR-LON-R10 (Anstee & Edwards, 1982) were chosen, the former because of its panepithelial staining and its role in clearing metastatic breast carcinoma cells from human bone marrow (Buckman *et al.*, 1982); this antibody was used at a concentration of 1–2 $\mu$g/200 $\mu$l of medium. LICR-LON-R10 was used as mouse ascitic fluid at a dilution of 1/100 in medium. This antiserum reacts with red cell glycophorin and was used as a control. Normal non-immunised rabbit serum at a dilution of 1/100 in medium was used as a negative control for the studies with polyclonal antisera.

[$^{51}$Cr]-sodium chromate labelled human breast carcinoma cells were used as target cells (MDA-MB-231; Cailleau *et al.*, 1974).

HPBL effector cells were obtained from human volunteers and bone marrow effector cells from normal human donors. The blood or bone marrow was diluted 1:3 in PBS, carefully layered onto Ficoll-Hypaque, density 1.077 and 1.100 respectively and centrifuged at 500 *g* for 20 min. The interface was collected, washed with medium and the cells suspended in an appropriate volume of medium.

The ADCC assay was performed by pipetting labelled target cells into 96-well U-bottomed plates (Microtitre M24 AR) at a concentration of $2\times10^4$ cells per 50 $\mu$l of medium per well, adding 100 $\mu$l of antibody and incubating for 1 h at 4°C. The cells were then centrifuged at 500 *g* for 5 min and the supernatants removed. Different concentrations of effector cells in 100 $\mu$l of medium were then added to each well to give effector cell:target cell ratios from 1:1 to 200:1. The plates were then incubated for 4 h at 37°C. After this time 50 $\mu$l of medium was added to each well to disturb the cells. The plates were then spun at 500 *g* for 10 min and 120 $\mu$l of supernatant removed and counted in a gamma counter. Maximum $^{51}$Cr-release was obtained by incubating target cells with 1% NP40. Percentage specific lysis was calculated in the usual way using

Correspondence: M.E. Gore

Received 2 June 1983; accepted 6 September 1983.

the formula:

%specific lysis =

$$\frac{\text{experimental release–spontaneous release}}{\text{maximum release–spontaneous release}} \times 100$$

The ability of LICR-LON-Fib-75 to mediate ADCC was investigated at a variety of effector cell:target cell (EC:TC) ratios using HPBL. Anti-EMA was used concurrently at the same EC:TC ratio in each experiment as a positive control (Table I). Maximum lysis achieved with LICR-LON-Fib-75 was 45% at an EC:TC ratio of 100:1. In the same experiment at the same EC:TC ratio anti-EMA produced 61% lysis.

**Table I** ADCC mediated by HPBL with anti-EMA or LICR-LON-Fib-75[a]

| *Effector cell: target cell ratio* | *Percentage specific lysis (±s.d.) using:* Anti-EMA | *LICR-LON-Fib-75* |
|---|---|---|
| 10:1 | 15.6±2.5 | 4±2.8 |
| 20:1 | 38.5±2.1 | 8±1.4 |
| 40:1 | 39±1 | 9.7±1.5 |
| 50:1 | 58±12.8 | 38.3±5.9 |
| 80:1 | 66±2.8 | 32±8.4 |
| 100:1 | 61±6.1 | 45.1±2.3 |
| 130:1 | 65±3.1 | 8.5±1.9 |

[a]Each estimation performed in triplicate.

Mean spontaneous lysis was 10.6±2.9%: controls were as follows: Target cells (TC) and anti-EMA (4.7±4.5%); TC and LICR-LON-Fib-75 (0.57±0.97%); TC and HPBL (2.7±1.8% at EC:TC 40:1; 1.8±2.5% at EC:TC 130:1).

Neither anti-MCF-7, LICR-LON-R10 nor normal non-immunised rabbit serum mediated ADCC at EC:TC ratios of 50:1, 50:1 and 20:1 respectively when HPBL were used.

The ability of LICR-LON-Fib-75 and anti-EMA to mediate ADCC using normal human marrow as a source of effector cells was investigated (Table II). Anti-EMA and LICR-LON-Fib-75 were run concurrently at the same EC:TC ratio. LICR-LON-Fib-75 and anti-EMA were also used with HPBL in each experiment as a positive control for the assay. When anti-EMA was used with bone marrow a small lytic effect was demonstrated. Although the percentage lysis was low (25% at the highest ET:TC ratio) this was above any control wells (bone marrow and target cells without antibody). It can be seen from Table II that bone marrow did not effect ADCC nearly as well as HPBL. In the same experiments LICR-LON-Fib-75 failed to produce any significant lysis above background levels when bone marrow was used as the source of effector cells.

HPBL were obtained from each of the bone marrow donors at the time of their marrow harvest and assayed for their ADCC effect; the percentage specific lysis was comparable to that achieved with HPBL from normal volunteers.

We have found that anti-EMA is a potent mediator of ADCC when HPBL are used as the source of effector cells. Although the monoclonal antibody LICR-LON-Fib-75 will also effect ADCC when HPBL are used, it does so to a lesser extent. Anti-EMA and not LICR-LON-Fib-75 will effect ADCC above control levels, when bone marrow is used as the source of effector cells. These results indicate that the failure of an antibody to mediate

**Table II** ADCC mediated by HPBL or bone marrow with anti-EMA or LICR-LON-Fib-75[a]

| *Effector cell: target cell ratio* | *Anti-EMA (HPBL)* | *Percentage specific lysis (±s.d.) using:* Anti-EMA (bone marrow) | *LICR-Fib-75 (HPBL)* | *LICR-Fib-75 (bone marrow)* |
|---|---|---|---|---|
| 6:1 | ND | 5.6±1.5 | 4.5±2.1 | 0.4±0.2 |
| 20:1 | 38.5±2.1 | 6±1 | 8±1.4 | 1±1 |
| 66:1 | 66±2.8 | 6.5±7.8 | 32±8.5 | 6±1.4 |
| 100:1 | 61±6.2 | 18±2.2 | 45.1±2.3 | 9.3±2.3 |
| 200:1 | ND | 25.7±4.3 | ND | 12.5±2 |

[a]Each estimation performed in triplicate.

ND = not done.

Mean spontaneous lysis was 8.5±1.4%: controls as follows: Target cells (TC) and anti-EMA (0%); TC and LICR-LON-Fib-75 (2%); TC and HPBL (0%); TC and bone marrow (1.5±0.7% at EC:TC 66:1; 2.6±0.1% at EC:TC 100:1; 14.9±0.1% at EC:TC 200:1).

ADCC with bone marrow is partly a function of the antiserum used. However, as all assays were terminated at 4 h, there remains the possibility that the difference in effector capability between bone marrow and peripheral blood lymphocytes is a function of reaction kinetics. This has not been investigated.

Nevertheless, the results obtained using LICR-LON-Fib-75 are of particular interest since this is the first monoclonal antibody which has been shown to be active in an ADCC assay where *human* effector cells have been used against *human* tumour cell targets and a reasonably high percentage specific lysis achieved. Previous attempts to demonstrate ADCC in such a system have not been successful (Nadler, Stashenko *et al.*, 1980; Dillman, Sobol *et al.*, 1982).

Mouse bone marrow-derived effector cells have been shown to mediate ADCC against red blood cell targets (Berger and Amos, 1977) and tumour cells (Haskill & Fett, 1976; Bursuker *et al.*, 1982), but it has not previously been shown that human bone marrow can be active in an ADCC assay against human tumour cells. The clinical significance of this activity is highly speculative but may merit further investigation particularly when treatments aimed at clearing malignant cells from bone marrow, by systemic or *in vitro* monoclonal antibody therapy, are proposed.

We would like to thank Dr. M.G. Ormerod, Institute of Cancer Research, for providing the antibody anti-EMA, Drs. C.S. Foster and P.A.W. Edwards for LICR-LON-Fib-75 and Dr. R.A.J. McIlhinney and Mr. S. Patel for its purification. We are grateful to Miss J. Pelly for her technical assistance with the cell line.

**References**

ANSTEE, D.J. & EDWARDS, P.A.W. (1982). Monoclonal antibodies to human erythrocytes. *Eur. J. Immunol.*, **12**, 228.

BERGER, A.E. & AMOS, D.B. (1977). A comparison of antibody-dependent cellular cytotoxicity (ADCC) mediated by murine and human lymphoid cell populations. *Cell. Immunol.*, **33**, 277.

BERSTEIN, I.D., TAM, M.R. & NOWINSKI, R.C. (1980). Mouse leukaemia therapy with monoclonal antibodies against a thymus differentiation antigen. *Science*, **207**, 68.

BUCKMAN, R., McILHINNEY, R.A.J., SHEPHERD, V., PATEL, S., COOMBES, R.C. & NEVILLE, A.M. (1982). Elimination of carcinoma cells from human bone marrow. *Lancet*, **ii**, 1428.

BURSUKER, I., GOLDMAN, R., SCHADE, U. & LOHMANN-MATTHES, M.L. (1982). Differences in antibody-dependent cellular cytoxicity against tumour cells in *in vitro* differentiating mononuclear phagocytes from bone marrows of normal and inflamed mice. *Cell Immunol.*, **68**, 53.

CAILLEAU, R., YOUNG, R., OLIVE, M. & REEVES, W.J. (1974). Breast tumour cell lines from pleural effusions. *J. Natl Cancer Inst.*, **53**, 661.

DEARNALEY, D.P., SLOANE, J.P., ORMEROD, M.G. & 7 others. (1981). Increased detection of mammary carcinoma cells in marrow swears using antisera to epithelial membrane antigen. *Br. J. Cancer*, **44**, 85.

DILLMAN, R.O., SOBOL, R.E., COLLINS, H., BEAUREGARD, J. & ROYSTON, I. (1982). In: *Hybridomas in Cancer Diagnosis and Treatment*. (Eds. Mitchell & Oettgen). New York: Raven Press, p. 151.

EDWARDS, P.A.W., FOSTER, C.S. & McILHINNEY, R.A.J. (1980). Monoclonal antibodies to teratomas and breast. *Transpl. Proc.*, **12**, 398.

HASKILL, J.S. & FETT, J.W. (1976). Possible evidence for antibody-dependent macrophage cytotoxicity against murine adenocarcinoma cells *in vivo*. *J. Immunol.*, **117**, 1992.

HERLYN, D., HERLYN, M., STEPLEWSKI, Z. & KOPROWSKI, H. (1979). Monoclonal antibodies in cell-mediated cytotoxicity against human melanoma and colorectal carcinoma. *Eur. J. Immunol.*, **9**, 657.

HERLYN, D.M., STEPLEWSKI, Z., HERLYN, M.F. & KOPROWSKI, H. (1980). Inhibition of growth of colorectal carcinoma in nude mice by monoclonal antibody. *Cancer Res.*, **40**, 717.

KIRCH, M.E. & HAMMERLING, U. (1981). Immunotherapy of mouse leukaemias by monoclonal antibodies. 1. Effect of passively administered antibody on growth in transplanted tumour cells. *J. Immunol.*, **127**, 805.

NADLER, L.M., STASHENKO, P., HARDY, R., KAPLAN, W.D., BUTTON, L.N., KUFE, D.W., ANTMAN, K.H. & SCHLOSSMAN, S.F. (1980). Serotherapy of a patient with a monoclonal antibody directed against a human lymphoma-associated antigen. *Cancer Res.*, **40**, 3147.

SLOANE, J.P. & ORMEROD, M.G. (1981). Distribution of epithelial membrane antigen in normal and neoplastic tissues and its value in diagnostic tumour pathology. *Cancer*, **47**, 1786.